# 

**Published:** 1864-10

**Authors:** 


### CONTENTS OF THE OCTOBER NUMBER.

PAGE.

ORIGINAL COMMUNICATIONS.

Report on Orthopedic Surgery, made at the Annual Meeting of the Illinois State Medical Society, convened in Chicago, May 3d, 1864. By David Prince, M. D., Jacksonville, Ill. .... 431

Clinical Lectures on Diseases of the Eye. By E. L. Holmes, M. D., of Chicago, Lecturer, on Diseases of the Eye and Ear in Rush Medical College—The Iris—Anatomy and Physiology .... 443

SELECTED.

On Certain Abuses of Caustics. By Dr. James Morton, Lecturer on Materia Medica, Anderson's University, etc .... 448

On the Hypodermic Treatment of Uterine Pain. By J. Henry Bennet, M. D., Late Physician Accoucheur to the Royal Free Hospital .... 452

Utility of Aloes in the Treatment of Wounds .... 456

Puerperal Convulsions Successfully Treated by Subcutaneous Injections of Morphia. By Prof. Scanzoni, of Wurtzburg .... 459

The La Pommerais Case .... 462

The Calabar Bean .... 463

On the Treatment of Albuminuria in Children .... 467

On Spurious Diphtheria; Its Nature and Treatment .... 470

Weight of the Lungs. By H. Allen, M. D., Asst. Surg. U. S. A. .... 472

Foreign Bodies in the Ear .... 473

Miscellaneous .... 474

